# Diseases of the Blood

**Published:** 1905-07-22

**Authors:** 


### PROGRESS IN MEDICINE AND SURGERY.

### DISEASES OF THE BLOOD.

(*Continued from page* 277.)

**Acute Leukæmia in Early Life.**—Churchill[7] has made a study of a case of acute leukæmia in a child aged four years, and of previous cases reported in medical literature. He concludes that tuberculosis does not play any part in causing the condition. Nothing seems to act as a predisposing cause, except, possibly, chronic tonsillitis; and it is probable that this is a result rather than the cause of the process. It is not improbable that the infectious agents found in the late stages gain access to the system *viâ* the tonsils. The general course of the disease in children, on the whole, is the same as in adults, and it is always fatal. The spleen is enlarged in all cases and the liver usually. General glandular involvement is by no means constant, the cervical glands being most frequently involved. The duration is from four days to five months. The onset, however, is usually so insidious that it is impossible to determine exactly when a case does begin. The acute part of the disease is undoubtedly but the final stage of a chronic process. The duration does not depend on the type of the disease. The most prolonged case, five months, was lymphocytic, whereas the one myelocytic case lasted two and a half months. There is always a diminution in erythrocytes and hæmoglobin. The leucocyte count varied from 6,000 to 810,000. A falling white count is apparently indicative of approaching death. The differential count showed that of 27 cases 25 were of the lymphocyte type, one myelocytic, and two of the mixed cell variety. The lymphocytosis is more apt to be of the small cell variety. This cell predominated in 12 cases, the large cell in three, and the two varieties were equal in two. The polymorphonuclears are always reduced, often to an extreme degree. Except in the myelocytic and mixed cases, myelocytes were rarely present, and then only in small numbers. Nucleated reds were also rare, though found occasionally, especially in young subjects. Certain minor differences in the behaviour of the disease in early life are observed; hæmorrhages are less frequent and less severe. They occur in the nose, mouth, bowels, kidneys, and skin. The blood-picture also presents a greater variety than in adults, and the lymphocytosis is more apt to be of the small cell type. In infancy profound anæmia and leucocytosis are so frequent and arise from such a variety of causes that error is easily made in diagnosis unless a differential white count is made, and, owing to the ease with which a lymphocytosis is set up, a constant and high lymphocyte count must be found to establish the diagnosis. Many researches have shown that the bone-marrow only need be involved in order to produce the disease, whether of the myelocyte or lymphocyte type; for the bone-marrow is always involved, the glandular system sometimes not at all. All cases are thus "myelogenous," and the varieties should be termed "myelocytic" and "lymphocytic," according to the presence of either myelocytes or an excess of lymphocytes in the circulating blood. Streptococci, staphylococci, and other organisms have been found in the blood, but these organisms are secondary invaders only and are not the causes of the condition, being found in late stages only. Treatment is of no avail; arsenic is useless, and the record of cases treated by *x*-rays gives no promise of permanent help.

**Chloroma.**—Dock and Warthin[8] have recorded a case of this disease, and have analysed all the cases of this rare disease which have been recorded since 1893. They summarise their conclusions as follows:—Chloroma is a tumour like hyperplasia of the parent cells of the leucocytes, primarily in the red marrow, the periosteum being involved only secondarily. As a result of this, a typical leucocytes, or leucocytes corresponding to one of the normal types, may appear in the blood in varying number. The disease must therefore be classed with the leukæmias. The leucocytes may be of the large lymphocyte type, or of the type of neutrophile or eosinophile myelocytes. Whether a small lymphocyte type exists remains to be seen. The blood picture may therefore be very varied, and if this is used for a basis of classification it is possible to designate different varieties of chloroma. It is also possible that in certain cases, or possibly stages, of chloroma the hyperplasia of the parent cells may reach such a stage that the new forms do not get into the blood-stream in such numbers as to give the picture of a leukæmia. An aleukæmic chloroma is therefore possible. The essential difference between chloroma and other forms of leukæmia is the more marked neoplastic character of the former with the formation of the green masses and the metastases. The white cells resulting from the atypical proliferation have the power of multiplying in the blood stream, thus giving rise to masses of cells having the characteristics of metastatic tumours. It is probable that the proliferative activity is proportionate to the lack of differentiation. The hyperplasia of certain of the elements of the bone-marrow leads to the crowding out and replacement of the erythroblastic elements. Chloroma is therefore the cause of a severe anæmia, which is one of deficient hæmatopoiesis, and not one of excessive hæmolysis. The malignancy of the disease depends chiefly on the replacement of the red-marrow and consequent anæmia. The slight antagonism between the new cells and the body cells would otherwise place it among the relatively benign growths. The cause of the green colour is unknown, and it is possible that processes morphologically like chloroma may lack the characteristic green colour. The ultimate etiology of the disease is unknown. We are in the same position here as in the case of neoplasms in general.

**The Diagnostic Value of Leucocytosis** is discussed in a paper by McCaskey,[9] who gives the following summary of his conclusions. A single count is quite insufficient, it should be followed up by several made under different conditions. An increase beyond ten or twelve thousand in the peripheral blood indicates varying degrees of intoxication, with chemotactic substances of some sort or another. Whether it indicates suppuration or not is a question to be determined by weighing all the facts in each case. The leukocytes indicating suppuration are of the neutrophile type. The eosinophile form of leucocytosis indicates cutaneous or parasitic diseases in the intestine or elsewhere. Lymphocytosis signifies an irritative lesion of the lymphatic apparatus. In the diagnosis of malignant disease a leucocytosis is of very subordinate value, and when present is probably not due to the malignant disease *per se*, but to co-existing chemotactic toxins. Drysdale,[10] in a discussion on the same question, said that observers should be on their guard against leucocytosis occurring under physiological conditions such as pregnancy, parturition, childhood, and digestion. The causes of leucocytosis might be grouped under inflammatory, toxic, cachectic and anæmic (especially post-hæmorrhagic). In the detection of appendicitis leucocytosis was of value, but not in its diagnosis from pyosalpinx. Pus might be present without leucocytosis. Watson said that it was a diagnostic fact of value that in tuberculous peritonitis there should be no leucocytosis, while in septic peritonitis it was very marked.

[7] Amer. Jour. Med. Sc., October. [8] Trans. Assoc. Amer. Phys., 1904. [9] Amer. Jour. Med. Sc., December. [10] Lancet, Oct. 29.

